# Knowledge and attitude of reproductive age group (15–49) women towards Ethiopian current abortion law and associated factors in Bahir Dar city, Ethiopia

**DOI:** 10.1186/s12905-020-00958-y

**Published:** 2020-05-06

**Authors:** Getasew Mulat Bantie, Amare Alamirew Aynie, Mihret kassa Assefa, Ayele Semachew Kasa, Tigabu Birhan Kassa, Gebiyaw Wudie Tsegaye

**Affiliations:** 1 Public Health Department, GAMBY College of Medical Sciences, Bahir Dar, Ethiopia; 2 GAMBY General Teaching Hospital, Bahir Dar, Ethiopia; 3 Department of Adult Health Nursing, College of Medicine and Health Sciences, Bahir Dar University, Bahir Dar, Ethiopia; 4 Bahir Dar Health Science College, Bahir Dar, Ethiopia; 5 School of Public Health, Bahir Dar University, Bahir Dar City, Ethiopia

**Keywords:** Abortion, Ethiopian abortion law, Reproductive-age women, Bahir Dar city

## Abstract

**Background:**

Unsafe abortion accounts for nearly 60% of all gynecologic admissions and almost 30% of all obstetric and gynecologic admissions. Studies on abortion in Ethiopia have given less attention to women’s perceptions and experiences of abortion laws. Although the 2005 revised abortion law allows women to access safe abortion services, still unsafe abortion is one of the leading causes of pregnancy-related deaths. Therefore, the current study aimed to assess women’s knowledge and attitude towards the Ethiopian current abortion law in Bahir Dar City Administration.

**Methods:**

A community-based cross-sectional study using a systematic random sampling technique was carried out among 403 randomly selected reproductive age women using a pre-tested structured questionnaire in Bahir Dar City Administration from May to June /2017. Data were entered into Epi data version 3.1 and analyzed using SPSS version 21.0 software. Logistic regression was done to identify the possible factors associated with women’s knowledge and attitude towards the Ethiopian current abortion law.

**Results:**

Three hundred eighty-six respondents partook with a response rate of 95.7%. The study showed that 43% had good knowledge and 38% had a favorable attitude towards the Ethiopian current abortion law. Women’s in the age group of 25–29 years (AOR = 2.7, 95% CI: 1.02, 6.9), partner’s educational status of primary (AOR = 2.9, 95% CI: 1.19, 7.08), secondary (AOR = 5.5, 95% CI: 2.09, 14.4) and college and above (AOR = 8.2, 95% CI: 2.3, 28.6) were significantly associated with good knowledge of the Ethiopian current abortion law. While partner’s educational status; college and above (AOR = 6.15, 95% CI: 1.87, 20.22) was significantly associated with the favorable attitude towards the Ethiopian current abortion law.

**Conclusions:**

43% of respondents had good knowledge and 38% had a favorable attitude towards the Ethiopian current abortion law. Forty-nine respondents had a history of abortion of which, 8 occurred through induction. Woman’s age and partner’s education determine the status of knowledge while merely; the partner’s educational status of college and above was significantly associated with the attitude towards Ethiopian current abortion law, respectively.

## Background

Abortion is a worldwide event affecting every country. In countries that have decriminalized abortion, women are spared of the dire consequences of illegal abortion. In many other countries where abortion is a criminal act, illegal abortion is the major cause of maternal mortality and other serious health problems [1].

The World Health Organization (WHO) determined that about 22 million unsafe abortions happen per year worldwide, nearly all of this happens in low-income countries. Complications from unsafe abortion result in maternal deaths and abortion-related morbidity worldwide, placing high strain on limited health system resources and leading to severe physical, psychological, and financial consequences for women [2, 3].

It is estimated that annually 2 to 4.4 million abortions among adolescents occur in developing countries. According to hospital records of many developing countries, between 38 and 68% of women treated for complications of abortion are under 20 years of age [4]. According to the Ethiopian Ministry of Health, complications related to unsafe abortion are the second leading cause of death for women after tuberculosis [5].

In the preceding Ethiopian criminal code, abortion was solely permitted to save the woman’s life. However, the new Ethiopian liberated (the 2004 revised), criminal code authorizes abortion without proofing the age of the mother and the situation of pregnancy when there is raped or incest pregnancy, maternal or fetal life threatened, severe fetal abnormalities, maternal physical or mental disabilities, the women physically or psychologically unprepared to raise the child happens [6]. Though this liberated law allows women to access safe abortion services, still unsafe abortion is one of the leading causes of pregnancy-related deaths.

About 60% of gynecology and 30% obstetric inpatient services were due to unsafe abortion. A study in Ethiopia revealed that 54% of maternal deaths caused from unsafe abortion [5]. There is a claim that this study finding is underestimated of the maternal death, as the unsafe abortion services are mostly undisclosed, and full of complications thought the complications from the unsafe abortion don’t come to health institutions.

Unsafe abortion is the process of interrupting the unwanted conception either by unskilled and uncertified individual or in an area lacking the standards for medical procedures or both. It is also under sub-optimal care of the complications of spontaneous abortion, WHO [7–9]. From the start of the twentieth century Abortion, laws have been released from prohibition when the magnitude of unsafe abortion acknowledged as the public health problems [10].

Studies in different parts of Ethiopia revealed that age, sex, occupation, and monthly income were the factors associated with knowledge towards the legalization of abortion. Similarly, religion, marital status, educational status, history of induced abortion, knowledge of legalization of abortion, preference of abortion if the pregnancy is unwanted, knowledge about complication of abortion and legalization of abortion would reduce associated complication were the factors associated with attitudes towards the legalization of abortion [11–13].

Knowing the level of knowledge and attitude of women of reproductive age group (15–49 years) towards the Ethiopian current abortion law is very important to correct the knowledge about abortion legal, law, to make women’s access to legal abortion service, to save women’s lives in case of rape, incest, and medical illness, to know the applicability of the law and to decrease the complication and death of women related to unsafe abortion. Therefore, the current study designed to assess women’s knowledge and attitude of the Ethiopian current abortion law in Bahir city.

## Methods

### Study design, setting and period

This study was employed in Bahir Dar City Administration from May 11 to June 18 / 2017. The Bahir Dar City is the capital city of Amhara National, regional state, Ethiopia. The city is administratively divided into nine sub-cities: Sefene Selam, Gish Abay, Fasilo, Belay Zeleke, Hidar 11, Shum Abo, Shimbit, Ginbot Haya and Tana.

### Study design

Community-based cross-sectional study design.

### Sample size determination and sampling procedure

The sample size was calculated by a single population proportion formula by the assumption that a 95% confidence interval (Z = 1. 96), 5% margin of error and 50% proportion. Finally, by adding a 5% non-response rate, 403 women sample size was determined.

$$ n=\frac{{\left( Za/2\right)}^2\ast p\ \left(1-p\right)}{d^2} $$

**Fig. 1:**
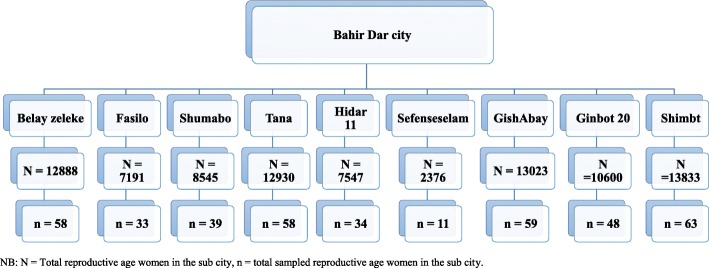
Schematic presentation of sampling procedures of reproductive age women towards current abortion law, Bahir Dar City Administration, 2017

To get the expected sample size, the lists of reproductive age group women in each sub-city were proportionally allocated using a systematic random sampling technique. Then, each reproductive age woman from each selected household was interviewed. When more than one eligible woman is available in the selected household, the simple random sampling method was employed. However, mentally and seriously ill reproductive-age women were excluded from the study (Fig. 1).

### Data collection process

The data were collected at the household level using Amharic (the local and national language of Ethiopia) version interviewer-administered structured questionnaire. The questionnaire was first developed in the English language by reviewing different kinds of literature [5–7, 11–13]. Then, back translated to the Amharic language. The questionnaire had four components; socio-demographic, obstetrics, knowledge as well as attitudes assessing questions. All the enumerators collected the data after having verbal consent from participants. To assure the quality of the data and to make sure that all assessment team members were able to administer the questionnaires properly, a one-day rigorous training was given to four public health graduated enumerators (all bachelor holders) and one master holder supervisor.

Data collectors and the supervisor carried out role-play practices and then had field pre-test activities in 5 % of the total sample size before the actual data collection. At the end of every data collection day, the supervisor examined each questionnaire and gave pertinent feedback to the data collectors. The internal consistency (Cronbach alpha) level of the pretest of knowledge and attitude assessing characteristics was between 0.76 and 0.82, respectively.

### Operational definition

A woman was considered as having good knowledge about the Ethiopian current abortion law when she responded ‘yes’ to the knowledge questions above the mean score. Otherwise, she was considered as having poor knowledge.

A woman was considered as having a favorable attitude towards the Ethiopian current abortion law when she responded ‘yes’ to the attitude questions above the mean score. Otherwise, she was considered as having an unfavorable attitude.

### Data analysis procedures

Data entry, data cleaning, and coding were performed using SPSS version 20 and analyzed with the same software. To explain the study population about relevant variables, frequencies and summary statistics was used. Predictor variables having a *p*-value < 0.20 were taken into a multivariable logistic regression analysis to see associations between dependent and independent variables. *P*-values less than 0.05 were taken the identified predictors in all cases.

### Ethical consideration

Ethical approval was obtained from GAMBY College of Medical Sciences, Research and Publication Office and approval letter were obtained from Bahir Dar City Administrative Office. The college ethics committee approved the procedure for verbal consent as the study is not a sensitive and privacy issue, rather assessing the familiarity and perceptions towards the Ethiopian current abortion law. Then, for 15–17 years old women, the verbal assents from them only with parental/guardian permission were secured. For > 17 years women, consent was secured solely from them. The objective of the study was clarified and informed verbal consent was received from the study participants to confirm their willingness. The respondents were notified that they have the right to refuse or terminate the study at any point of the interview. Because we obtained verbal consent, documentation of consent was not required. However, the information provided by each respondent was kept confidential in a secure place.

## Results

### Socio-demographic characteristics

Among the total 403 women of reproductive age group (15–49 years), 386 study participants participated in this study yielded a response rate of 95.7%. Of these, 47.15% (182) were aged 24 years and under, 20.73% (80) were in the age group of 25–29 years, 12.18% (47) were in the age group of 30–34 years and 19.9% (77) were in the age range of 35 years and above. Regarding their level of education, 17.37% (67) of the respondents could not read and write, 23.83% (92) attended primary school, 31.61% (112) attended secondary school and 27.2% (105) were college and above. Concerning the participants’ religion, 85.5% (330) were Orthodox Christian and 11.4% (44) were Muslim. Regarding occupation, 28.24% (109) respondents were students, 26.17% (101) were housewives and 25.17% (98) were private employees. The monthly income of 74.87% (289) respondents were ≤ 74 USD (Table 1).

**Table 1:** Socio-demographic characteristic of reproductive age women towards Ethiopian current abortion law in Bahir Dar city, northwest Ethiopia, 2017

Variable	Categories	Frequency	Percentage
Age	< 24	182	47.15
	25–29	80	20.73
	30–34	47	12.18
	> 35	77	19.9
Level of education	Unable to read and write	67	17.34
	Primary	92	23.83
	Secondary	122	31.61
	College and above	105	27.2
Religion	Orthodox	330	85.5
	Muslim	44	11.4
	Catholic	2	0.52
	Protestant	6	1.55
	Other	4	1.04
Occupation	Student	109	28.24
	Government employee	78	20.21
	Private employee	98	25.39
	House Wife	101	26.17
Current marital status	Single	180	46.6
	Married	176	45.6
	Divorced	18	4.7
	Widowed	12	3.1
Partner education level	Unable to read and write	23	13.1
	Primary	41	23.3
	Secondary	50	28.4
	college and above	62	35.2
Partner occupation	Student	4	2.3
	Government employee	69	39.2
	Private employee	90	51.1
	Merchant	13	7.4
Monthly income (in Ethiopian Birr)	< 74 USD	289	74.87
	74.1–148.1 USD	65	16.84
	> 148.2 USD	32	8.29
	**Total**	**386**	

Other: Wahhabi, Atheist, USD: United States Dollar

### Obstetric characteristics

Of the total 386 study participants, 179 had a history of pregnancy. Regarding gravidity, twenty-two respondents had got pregnant more than five times. Seventy-one participants got pregnant for the first time at the age of 19 years old, while 50 gave birth for the first time at the age younger than 19 years old. The study revealed that 49 respondents had a history of abortion. From whom, 10 respondents had 2–4 times abortion history and all of them were induced abortion. Among the 8 women that had induced abortion, 2 were at home and 6 in health clinics. The main reasons for seeking induced abortion were being unmarried, being raped, due to economic problems and being a student, respectively (Table 2).

**Table 2:** Obstetric characteristic of reproductive age women towards Ethiopian current abortion law in Bahir Dar city, northwest Ethiopia, 2017

Variable	Category	Frequency	Percentage
Have you ever been pregnant?	Yes	179	46.37
	No	207	53.62
Gravidity	1	65	36.3
	2–4	92	51.4
	> 5	22	12.3
Age at first pregnancy	< 19	71	39.7
	20–24	72	40.2
	> 25	36	20.1
Age at first birth	< 19	50	27.9
	20–24	90	50.3
	> 25	39	21.8
Abortion history	Yes	49	27.37
	No	130	72.63
Number of abortions	1	39	79.6
	2–4	10	20.4
Abortion type	Spontaneous	39	79.6
	Induced	8	16.3
	Both	2	4.1
Reason for inducing abortion	Economical	2	25
	Unmarried*	3	37.5
	Rape	2	25
	being student	1	12.5
Place where the induced abortion done	Home	2	25
	Health institute	6	75

Unmarried* = those respondents who currently not formally married (friendship, divorced, widowed)

### Knowledge concerning characteristics

Sixty-eight percent of the reproductive age women heard about the Ethiopian current abortion law. Regarding their knowledge level, considering seven knowledge, assessing questions, 43% had good knowledge about the Ethiopian current abortion law (Table 3).

**Table 3:** Knowledge related characteristics of reproductive age women towards Ethiopian current abortion law in Bahir Dar city, northwest Ethiopia, 2017

Variables	Category	Frequency	Percent
Have you ever heard about the current Ethiopian abortion law?	Yes	262	67.87
	No	124	32.12
The Ethiopian current abortion law permits for raping pregnant woman to terminate her pregnancy	Yes	220	56.99
	No	113	29.27
	Don’t know	53	13.73
The Ethiopian current abortion law permits to terminate pregnancy when a woman is endangered	Yes	199	51.55
	No	107	27.72
	Don’t know	80	20.72
The Ethiopian current abortion law permits to terminate pregnancy when her fetus is endangered	Yes	133	34.46
	No	146	37.82
	Don’t know	107	27.72
The Ethiopian current abortion law permits to terminate pregnancy when the woman is physically and psychologically unprepared	Yes	140	36.27
	No	175	45.34
	Don’t know	71	18.39
The Ethiopian current abortion law permits to terminate pregnancy when the woman’s age is < 18 years	Yes	229	59.33
	No	104	26.94
	Don’t know	53	13.73
The Ethiopian current abortion law permits to terminate pregnancy when the woman gets pregnant from her relatives	Yes	242	62.69
	No	96	24.87
	Don’t know	48	12.43
**The composite score of knowledge on** **Ethiopian current abortion law**	**Good knowledge**	**167**	**43.3**
	**Poor knowledge**	**219**	**56.7**

### Attitude characteristics

Taking eleven attitudes assessing questions into consideration, the composite score of the respondents having a favorable attitude to Ethiopian current abortion law was about 39%. Regarding the response to the question ‘do you think induced abortion should be legal’, about 58% of the study participants thought induced abortion shouldn’t be legalized (Table 4).

**Table 4:** Attitude related characteristics of reproductive age women towards Ethiopian current abortion law in Bahir Dar city, northwest Ethiopia, 2017

Variable	Category	Frequency	Percentage
Do you think abortion should be legal	Yes	161	41.71
	No	225	58.29
Do you think pregnancy should interrupt	Yes	148	38.34
	No	238	61.66
Do you think pregnancy occur from rape should be interrupted	Yes	162	41.97
	No	224	58.97
Do you think the pregnancy from relatives should be interrupted	Yes	159	41.97
	No	227	58.03
Do you think pregnancy should be interrupted when it endangers women and fetuses	Yes	147	38.08
	No	239	61.92
Do you think pregnancy should be interrupted by economically poor women	Yes	121	31.35
	No	265	68.65
Do you think pregnancy should be interrupted by age < 18 year	Yes	118	30.57
	No	268	69.43
Do you think pregnancy should be interrupted if fetus abnormality	Yes	133	34.46
	No	253	65.54
Do you think pregnancy should be interrupted mental abnormality	Yes	131	33.93
	No	255	66.06
Do you think pregnancy should be interrupted out of health institute	Yes	102	26.42
	No	284	73.58
Will you obey the abortion law when you are pregnant and wish to terminate a pregnancy	Yes	173	44.82
	No	213	55.18
**The composite score of attitude on Ethiopian current abortion law**	**Favorable attitude**	**149**	**38.6**
	**Unfavorable attitude**	**237**	**61.4**

### Factors associated with knowledge of the Ethiopian current abortion law

On bivariate analysis, women’s age, level of education, occupation and partner education were statistically associated with women’s knowledge status of the Ethiopian current abortion law, whereas in the multivariate analysis, women’s occupation and education did not show a significant association with knowledge status towards the Ethiopian current abortion law.

Those women between 25 and 29 years of age were about 2.65 times more likely to have good knowledge as compared to those whose age was greater than or equal to 35 years.

Regarding the partner’s education, the study revealed that as the educational status of their partner increases the likelihood of the women having good knowledge also increases (Table 5).

**Table 5:** Factors associated with knowledge of reproductive age women towards Ethiopian current abortion law in Bahir Dar city, northwest Ethiopia, 2017

Variable	Category	Knowledge of current Ethiopian abortion law		COR (95% CI)	AOR (95% CI)
		Good	Poor		
		n (%)	n (%)		
Women’s Occupation	House wife	32 (29.4)	77 (70.6)	**1.00**	**1.00**
	Private employee	24 (30.8)	54 (69.2)	1.06 (0.56,2.01)	
	Government employee	54 (55.1)	44 (44.9)	**2.95 (1.66,5.23)** ***	
	Student	57 (56.4)	44 (43.6)	**3.11 (1.76,5.51)** ***	
Women’s education	Unable to read and write	20 (65.7)	40 (34.3)	**1.00**	**1.00**
	Primary	44 (45.6)	94 (54.4)	0.93 (0.49,1.78)	
	Secondary	59 (32.8)	62 (67.8)	**1.90 (0.99, 3.62)**^¥^	
	College and above	44 (39)	23 (61)	**3.82 (1.83,7.99)** ***	
Women’s age	< 24 years	80 (44)	102 (56)	0.94 (0.551,1.60)	1.46 (0.57,3.73)
	25–29 years	40 (50)	40 (50)	1.20 (0.641,2.24)	**2.65 (1.02,6.89)** *
	30–34 years	12 (25.5)	35 (74.5)	**0.41 (0.186,0.91)**^¥^	0.68 (0.24,1.90)
	> 35 years	35 (45.5)	42 (54.5)	**1.00**	**1.00**
Partner’s education	Unable to read and write	14 (78.3)	48 (21.7)	**1.00**	**1.00**
	Primary	24 (65.9)	26 (34.1)	**3.16 (1.40,7.14)**^¥^	**2.91 (1.19,7.08)** *
	Secondary	27 (48)	14 (52)	**6.61 (2.74, 15.91)** ***	**5.48 (2.09,14.39)** **
	College and above	18 (22.6)	5 (77.4)	**12.34 (3.88,39.21)** ***	**8.15 (2.32,28.55)** **

Significant at ^¥^*p*-value < 0.2; * *p*-value < 0.05, ** *p*-value < 0.01, *** *p*-value < 0.001

### Factors associated with the attitude of Ethiopian current abortion law

On bivariate analysis, women’s level of education, women’s occupation, partner’s education, and partner’s occupation were statistically associated with attitude status towards the Ethiopian current abortion law, whereas in the multivariable analysis, only partner’s education had shown a significant association with women’s attitude towards the Ethiopian current abortion law.

Those women whose partners’ educational status were college and above were about 6.15 times more likely to have a favorable attitude towards the Ethiopian current abortion law as compared to those women whose partners’ educational level was characterized by the inability to read and write (Table 6).

**Table 6:** Factors associated with an attitude of reproductive age women towards Ethiopian current abortion law in Bahir Dar city, northwest Ethiopia, 2017

Variable	Category	Women’s Attitude towards current Ethiopian abortion law		COR (95% CI)	AOR (95% CI)
		Unfavorable	Favorable		
		n (%)	n (%)		
**Occupation**	Student	56 (72.4)	53 (27.6)	**2.46 (1.38, 4.38)** **	
	Government employee	37 (72.3)	41 (27.7)	**2.88 (1.55, 5.38)** **	
	Private employee	71 (51.4)	27 (48.6)	0.99 (0.53, 1.84)	
	House wife	73 (47.4)	28 (52.6)	**1.00**	**1.00**
**Woman’s Education**	Unable to read and write	33 (55)	27 (45)	**1.00**	**1.00**
	Primary	77 (55.8)	61 (44.2)	0.96 (0.52, 1.78)	
	Secondary	73 (60.3)	48 (39.7)	0.80 (0.43, 1.50)	
	College and above	54 (80.6)	13 (19.4)	**0.29 (0.13, 0.64)** **	
**Partner’s education**	Unable to read and write	55 (80.6)	28 (19.4)	**1.00**	**1.00**
	Primary	62 (61)	29 (39)	0.91 **(**0.48**,** 1.73)	1.53 (0.42, 5.58)
	Secondary	66 (55.8)	34 (44.2)	1.01 (0.54**,** 1.87)	2.23 (0.65, 7.65)
	College and above	54 (55.4)	58 (44.6)	**2.11 (1.17, 3.79)** *	**6.15 (1.87, 20.22)** **
**Partner’s occupation**	Student	2 (82.6)	2 (17.4)	**5.5 (0.46, 65.16)**^¥^	
	Government employee	37 (75.6)	32 (24.4)	**4.75 (1.98, 23.07)**^¥^	
	Private employee	61 (68)	29 (32)	2.61 (0.54,12.57)	
	Merchant	11 (43.5)	2 (56.5)	**1.00**	**1.00**

Significant at ^¥^*p*-value < 0. 2; * *p*-value < 0.05, ** *p*-value < 0.01, *** *p*-value < 0.001

## Discussion

Next to the 2005 revised abortion law, the Ethiopian Ministry of Health prepared the Technical and Procedural Guidelines for Abortion care in 2006. This guideline opening out a clue to a swift expansion of health facilities which providing safe abortion services through training of health professionals and making partnership with non-governmental organizations to advocate the new abortion law and to enhance and expand the provision of safe abortion services to the community worldwide [14].

Lack of cognizance towards the current abortion law will impact reproductive-age women’s decision-making ability for accessing and utilizing available services in receiving abortion care. Again, this may lead them to practice unsafe abortion. Recently, the Ethiopian Ministry of Health (MoH) has emphasized on the provision of information towards vital and safe abortion care. Because women’s awareness and service utilization who are living in areas of the country is low [15]. In this current study, 67.8% of the study participants heard about the Ethiopian current abortion law, whereas a study was done in Addis Ababa, Ethiopia revealed that the majority of the respondents were not aware of the Ethiopian current abortion law [13].

Moreover, our study is not consistent with a study done in Yirga Cheffee, Southern Ethiopia, which showed that 48.2% of the study participants had good knowledge and from these, 61.2% had a favorable attitude about legal laws and on the legalization of abortion in Ethiopia [12]. Another study was done in, Mizan Amman town, southern Ethiopia revealed that only 5.7% of respondents knew the current legal status of induced abortion in Ethiopia [11].

In comparison with a study done in Yirga Chefee, South Ethiopia, the current study participants had less awareness about the Ethiopian current abortion law. The present study showed 57% of the study participants knew that the Ethiopian current abortion law permits induced abortion when the pregnancy is in case of rape and 51.6% when the pregnancy endangers the life and health of the women and the fetus. Whereas a study was done in Yirga Chefee, South Ethiopia indicated that 86.2% of the participants knew that induced abortion is legalized in the case of rape and 60.1% when the pregnancy endangers the life and health of the woman and the fetus [12]. This shows that there is a discrepancy in awareness creation for women about the Ethiopian current abortion law from place to place due to different reasons. These could be due to low access to health education about the current abortion law to society, especially to women in the reproductive age group. This could be also due to the place where the study was done, religious aspect, social belief, low social media coverage, and many other reasons. Complications from unsafe abortion are very common among economically poor and younger age women. These women are the ones with limited access to family planning service and this leads them to have unwanted pregnancies. In addition to this group of women had limited access to available methods to abortion care [16].

The current study revealed that 49 respondents had a history of abortion of which, 8 occurred through induction. The main reasons for seeking induced abortion include being unmarried and being a student. This finding is consistent with a study done in southern Ethiopia [11].

In the present study, 6 (75%) of induced abortions were done at health facilities and this finding is almost consistent with different studies in Ethiopia [5, 11]. But in this study, 2 (25%) induced abortions were done at home using traditional medicine by mixing natural alcohol with different plant root extraction and decomposition. These harmful traditional and fatal practices might lead to severe maternal and fetal morbidity, like severe bleeding, loss of consciousness, organ failure. Consequently, might end up with mortality.

The present study showed that women’s age [25–29 years (AOR = 2. 7: 95%CI: 1.02,6.89), and partner’s educational status [Primary (AOR = 2.9: 95%CI: 1.2, 7.1), Secondary (AOR = 5.5: 95%CI: 2.1, 14.4), College and above (AOR = 8.1: 95%CI: 2.3, 28.6)] determine the status of knowledge while merely, partner’s educational status of college and above (AOR = 6. 1:95%CI: 1.9, 20.2) was significantly associated with attitude towards Ethiopian current abortion law, respectively. The finding is consistent with a study conducted in Addis Ababa, Ethiopia [13]. The discovery of this study revealed that the odds of a partner’s educational status positively influenced the likelihood of a woman’s knowledge and attitude towards the Ethiopian current abortion law. As the educational status of the partner’s advances, the probability of reproductive age women having good knowledge as well as favorable attitude increases. The possible justification for this typical finding might be because the culture believes that the local community adopted from their parents that perceived as ‘males are the superiors, and the females must expect everything from males’. However, when the partners educated more and more, the chance of avoiding these bad traditional believes will increase.

Accordingly, it might create a conducive environment for their couples to get involved in the open discussion on reproductive health and related legislation. Similarly, when the partners advanced in their education, the likelihood of seeking information and using electronic media will update; as a result, reproductive-age women might have better access to health information dissemination and might enhance their knowledge and attitude level towards Ethiopian current abortion law.

## Conclusion

Knowledge and attitude towards the Ethiopian current abortion law are low as per, the national standard. The study revealed that 43% of respondents had good knowledge and 38% had a favorable attitude towards the Ethiopian current abortion law. Forty-nine respondents had a history of abortion. The woman’s age and her partners education determine whether the woman is informed about the abortion law; favorable attitudes towards the law are related only to the partner having at least a college education. It is advisable to including men in current government efforts to educate the public about the abortion law.

## Supplementary information

**Additional file 1.** Questionnaire.

## Data Availability

The data can be accessed from the corresponding author upon justified request.

## Abbreviations

AOR: Adjusted odds ratio
BSc: Bachelor of Science
COR: Crude Odds Ratio
Epi Data: Epidemiological Data
MoH: Ministry of Health
MPH: Master of Public Health
SPSS: Statistical Package for Social Scientists
USD: United States Dollar
WHO: World Health Organization
