# Supplementary material for: Knowledge and attitude of reproductive age group (15–49) women towards Ethiopian current abortion law and associated factors in Bahir Dar city, Ethiopia
Source: BMC Womens Health. 2020 May 6;20:97. doi: 10.1186/s12905-020-00958-y (PMC7204207; doi:10.1186/s12905-020-00958-y)
Supplement: Supplementary file 1 — Additional file 1. Questionnaire. [file 12905_2020_958_MOESM1_ESM.docx]

Questionnaire

Part one: socio-demographic characteristics of the respondents

| **Characteristics** | **Response** | **Skip** |
| --- | --- | --- |
| Age (in years) | **______________** |  |
| Educational status | 1. Unable to read and write 2. Primary 3. Secondary 4. College and above |  |
| Religion | 1. Orthodox 2. Muslim 3. Catholic 4. Protestant 5. Others (if any specify) ____________ |  |
| Occupation | 1. Student 2. Government employee 3. Private employee 4. House Wife |  |
| Current marital status | 1. Single 2. Married 3. Divorced 4. Widowed |  |
| Partner education level | 1. Unable to read and write 2. Primary 3. Secondary 4. College and above |  |
| Partner occupation | 1. Student 2. Government employee 3. Private employee 4. Merchant |  |
| Monthly income (in Ethiopian birr) | ___________________ |  |

**Part Two: Obstetric related characteristics of the respondents**

| **Characteristics** | **Response** | **Skip** |
| --- | --- | --- |
| Have you ever been pregnant? | 1. Yes 2. No | If ‘No’ jump to |
| Number of live births | 1. One 2. Two up to four 3. Five and above |  |
| Age at first pregnancy (in years) | 1. <19 2. 20-24 3. >25 |  |
| Age at first birth (in years) | 1. <19 2. 20-24 3. >25 |  |
| Abortion history | 1. Yes 2. No |  |
| How many abortions | 1. One 2. 2-4 3. >5 |  |
| Abortion type | 1. Spontaneous 2. Induced 3. Both |  |
| Reason for induced abortion | 1. Economical 2. Unmarried 3. Rape 4. Being student |  |
| Place where the induced abortion done | 1. Home 2. Health institution |  |
| If you terminated your pregnancy at home, what did you used to terminate the pregnancy? | _______________________ |  |

**Part Three: Knowledge assessing characteristics of the respondents**

| **Characteristics** | **Response** | **Skip** |
| --- | --- | --- |
| Have you ever heard about the Ethiopian current abortion law? | 1. Yes 2. No |  |
| The Ethiopian current abortion law permits for raped woman to terminate her pregnancy | 1. Yes 2. No 3. Don’t know |  |
| The Ethiopian current abortion law permits to terminate pregnancy when a woman is endanger | 1. Yes 2. No 3. Don’t know |  |
| The Ethiopian current abortion law permits to terminate pregnancy when her fetus is endanger | 1. Yes 2. No 3. Don’t know |  |
| The Ethiopian current abortion law permits to terminate pregnancy when the woman is physically and psychologically unprepared | 1. Yes 2. No 3. Don’t know |  |
| The Ethiopian current abortion law permits to terminate pregnancy when the woman’s age is <18 years | 1. Yes 2. No 3. Don’t know |  |
| The Ethiopian current abortion law permits to terminate pregnancy when the woman get pregnancy from her relatives | 1. Yes 2. No 3. Don’t know |  |

**Part Four: Attitude assessing characteristics of the respondents**

| **Characteristics** | **Response** | **Skip** |
| --- | --- | --- |
| Do you think induced abortion should be legal? | 1. Yes 2. No |  |
| Do you think pregnancy should be terminated? | 1. Yes 2. No |  |
| Do you think pregnancy occur from rape should be terminated? | 1. Yes 2. No |  |
| Do you think pregnancy from relatives should be terminated? | 1. Yes 2. No |  |
| Do you think pregnancy should be terminated when it endangers the woman or fetus? | 1. Yes 2. No |  |
| Do you think pregnancy should be terminated when a woman is economically poor? | 1. Yes 2. No |  |
| Do you think pregnancy should be terminated when a woman is younger than 18 years old? | 1. Yes 2. No |  |
| Do you think pregnancy should be terminated if fetus has congenital abnormalities? | 1. Yes 2. No |  |
| Do you think pregnancy should be terminated mental abnormality? | 1. Yes 2. No |  |
| Do you think pregnancy should be terminated out of health institutions? | 1. Yes 2. No |  |
| Will you obey the abortion law when you are pregnant and wish to terminate a pregnancy? | 1. Yes 2. No |  |
